# A systematic review and meta‐analysis of the impact of the left atrial appendage closure on left atrial function

**DOI:** 10.1002/clc.23824

**Published:** 2022-04-02

**Authors:** Mostafa Reda Mostafa, Mohamed Magdi, Ahmad Al‐abdouh, Waiel Abusnina, Mostafa Elbanna, Basel Abdelazeem, Sarath Lal Mannumbeth Renjithal, Mamas A Mamas, Jaffer Shah

**Affiliations:** ^1^ Department of Internal Medicine Rochester Regional Health, Unity Hospital Greece New York USA; ^2^ Department of Medicine University of Kentucky Lexington Kentucky USA; ^3^ Department of Cardiology Creighton University School of Medicine Omaha Nebraska USA; ^4^ Department of Medicine Mayo Clinic Phoenix Arizona USA; ^5^ Department of Medicine McLaren Health System Flint Michigan USA; ^6^ Department of Cardiology University of Manchester Manchester UK; ^7^ New York State Department of Health New York USA

**Keywords:** amulet device, atrial function, left atrial appendage closure, left atrial emptying fraction, left atrium function, WATCHMAN device

## Abstract

**Background:**

Left atrial (LA) appendage closure (LAAC) is effective in patients with atrial fibrillation who are not candidates for long‐term anticoagulation. However, the impact of LAAC on LA function is unknown. The aim of this study is to evaluate the impact of LAAC on atrial function.

**Methods:**

This meta‐analysis was conducted according to Preferred Reporting Items for Systematic Reviews and Meta‐Analyses guidelines. A search strategy was designed to utilize PubMed/Medline, EMBASE, and Google scholar for studies showing the effect of LAAC on the LA function from inception to November 20, 2021. The standardized mean difference (SMD) was calculated from the means and standard deviations.

**Results:**

Of 247 studies initially identified, 8 studies comprising 260 patients were included in the final analysis. There was a significant increase in LA emptying fraction following LAAC compared with preoperative function (SMD: 0.53; 95% confidence interval [CI]: 0.04–1.01; *p* = .03; *I* ^2^ = 75%). In contrast, there were no significant differences in LA volume (SMD: −0.07; 95% CI: −0.82–0.69; *p* = .86; *I* ^2^ = 92%) peak atrial longitudinal strain (SMD: 0.50; 95% CI: −0.08–1.08; *p* = .09; *I* ^2^ = 89%), peak atrial contraction strain (SMD: 0.38; 95% CI: −0.22–0.99; *p* = .21; *I* ^2^ = 81%), strain during atrial contraction (SMD: −0.24; 95% CI: −0.61–0.13; *p* = .20; *I* ^2^ = 0%), strain during ventricular systole (SMD: 0.47; 95% CI: −0.32–1.27; *p* = .24; *I* ^2^ = 89%), strain during ventricular diastole (SMD: 0.09; 95% CI: −0.32–0.51; *p* = .66; *I* ^2^ = 65%).

**Conclusion:**

LAAC is associated with improvement in the left atrial emptying fraction, but did not significantly influence other parameters.

**Abbreviation**
Afib: atrial fibrillation
CABG: coronary artery bypass graft
CHF: congestive heart failure
CMR: cardiac magnetic resonance
FDA: Food and Drug Administration
HFpEF: heart failure preserved ejection fraction
HFrEF: heart failure with reduce ejection fraction
LA: left atrium
LAA: left atrial appendage
LAAC: left atrial appendage closure
LAEF: left atrial emptying fraction
LV: left ventricle
NVAF: nonvalvular atrial fibrillation
PACS: peak atrial contraction strain
PALS: peak atrial longitudinal strain
TAEF: total atrial emptying fraction

## 1 INTRODUCTION

Standard treatment in nonvalvular atrial fibrillation is oral anticoagulation, either direct oral anticoagulants or warfarin.^1^ For patients who are unable tolerate oral anticoagulant agents, or have a contra‐indication, left atrial appendage closure (LAAC) is considered an effective alternative with less risk of bleeding. The closure of the Left atrial appendage (LAA) has been approved for some devices. In 2015, the Food and Drug Administration authorized the WATCHMAN as the first device for LAA, and the AMPLATZER™ Amulet™ was approved in 2021.^2^

These devices prevent systemic embolization by closing off the LAA, which is considered the source of thrombus formation in more than 90% of the cases.^3^ The LAA is an actively contracting structure and more distensible than the rest of the atrium and should not be considered to be merely a superfluous appendix and a source of emboli.^4^ It is rich in stretch receptors and more sensitive to pressure fluctuation than the left atrial (LA).^5^ Also, the LAA has an essential endocrine function as it is a storage for the atrial and brain natriuretic peptides (BNPs).^6^ Those characteristics allow the LAA to play a role in the pressure regulation inside the left atrium and fluid balance by enhancing diuresis. Therefore, the LAAC may theoretically affect LA hemodynamics. We, therefore, studied the impact of LAAC on the LA hemodynamics through conducting a meta‐analysis of published literature.

## 2 METHODS

### 2.1 Data source and search strategy

The present meta‐analysis was performed in accordance with Preferred Reporting Items for Systematic Reviews and Meta‐Analyses guidelines and the Cochrane handbook.^7^

We systematically searched MEDLINE/PubMed, EMBASE, and Google scholar from inception to November 30, 2021. The search included the following key terms: ((“left atrial function”) AND (“left atrial appendage closure” OR “left atrial appendage exclusion”)) AND (“echocardiography” OR “speckle tracking echocardiography”). We further reviewed the references list of the included articles in this review to include other relevant studies.

### 2.2 Eligibility criteria

We included articles that demonstrate the impact of LAA exclusion on LA functions assessed by speckle tracking echocardiography. Articles using other methods other than speckle echocardiography for assessment were excluded from our analysis. In‐vitro studies, non‐English papers, reviews and data that could not be extracted were also excluded.

### 2.3 Outcome of interest, data extraction, and quality assessment

While the primary outcome in our study is to assess LA reservoir function and LA contractile function, the secondary outcome is to assess LA systolic function by strain rates. LA reservoir function is assessed by LAEF and peak atrial longitudinal strain (PALS), and LA contractile function is assessed by peak atrial contraction strain (PACS).

Initial title and abstract screening were conducted by two reviewers (M.R.M and M.M) and all disagreements were discussed to reach a consensus, otherwise, a third opinion from W.S was obtained.

Potentially eligible articles were imported for full‐text review and assessed for inclusion. We extracted data using an Excel sheet. Examples of data collected are sample size, mean of age, female gender %, CHA_2_DS_2_‐VASc score mean, hypertension (HTN) %, diabetes mellitus (DM) %, hyperlipidemia %, congestive heart failure %, coronary artery disease (CAD) %, prior ablation % and stroke %.

We assessed the quality of the included studies using the Newcastle–Ottawa Scale for cohort studies, as shown in Table S1. For Newcastle–Ottawa Scale, each asterisk counts as one point.^8^ The maximum points are two for comparability and one for all other categories (Table S1). Each star adds to the total score. A score of <5 is considered low quality, 5–6 is medium quality, while 7–9 is high quality. In the included studies, two were low quality, four were medium quality and one was of high quality. We did not perform funnel plots for publication bias since the number of the included studies is <10 in our analysis.^9^

### 2.4 Data synthesis and analysis

We used the random‐effects model with 95% confidence intervals (CIs) to calculate the standardized mean difference (SMD) from the means and standard deviations. The means and standard deviations were calculated as described by Wan et al.^10^ when unavailable in the selected studies. We used the *I* ^2^ to measure heterogeneity over the included studies (<25% considered low heterogeneity, and >50% considered significant heterogeneity.^11^ Analyses were performed using R Studio Version 3.6.3.

## 3 RESULTS

### 3.1 Summary of studies

**Figure 1:**
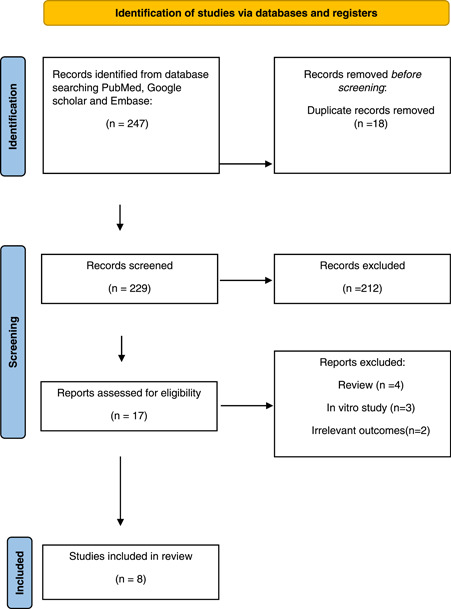
PRISMA. PRISMA, Preferred Reporting Items for Systematic Reviews and Meta‐Analyses

A total of 247 articles were screened from a comprehensive electronic database search. After thorough review, we found 8 retrospective or prospective observational studies (one of them was only published as an abstract) that evaluated the LA function after LAAC.^12^, ^13^, ^14^, ^15^, ^16^, ^17^, ^18^, ^19^ The search process is detailed in (Figure 1). The pertinent details of the included studies are illustrated in (Table 1). A total of 260 patients were included in our analysis from 8 studies and we compared LA performance preoperatively with its performance postoperatively. The baseline characteristics of patients included in our study are detailed in (Table 2).

**Table 1:** Summary of the included studies

Study	Year/country	Study design	Sample size	Follow‐up	Summary of methodology	Outcome
Madeira et al.^12^	2018/Portugal	Retrospective	16	N.A	TEE performed before and after LAAC. LA volumes were calculated using the biplane method, and LA mechanics were assessed using STE. The analysis focused on the LA reservoir phase strain and strain rate.	percutaneous LAA closure does not affect LA reservoir function
Yang et al.^13^	2021/China	Retrospective	65	12 months	Intervention group: Combined AF ablation and LAAC. Control group: AF ablation. echocardiography and speckle tracking echocardiography were performed to assess LA reservoir, conduit, and contractile function	Both the combined therapy group and the simple ablation group demonstrated significant improvement in LA function. But most of the effects appeared to result from ablation, not LAAC
Ijuin et al.^14^	2020/Germany	Retrospective	95	180 days	95 patients who underwent percutaneous LAAC. LA strain was evaluated at three different time intervals by TEE (baseline, 45 days, and 180 days after the procedure	The study showed improvement in TEE‐derived LA strain following LAAC within 45 days of implantation.
Coisne et al.^15^	2016/France	Prospective	33	45 days	33 patients evaluated by TEE at time of discharge and 45 days.	LAA closure was associated with an improvement in LA mechanical function
Murtaza et al.^16^	2019/USA	Prospective	25	N.A	25 patients underwent LAAC. LA function parameters (volumetric, strain indices) were assessed by speckle tracking before and after	LAAC leads to improvement in all volumetric indices of the LA. However, there was discrepancy between volumetric and strain indices
Sharma et al.^17^	2021/USA	Retrospective	67	N.A	TTE performed before and after LAAC.	LAAC resulted in elevated LV filing pressure, improvement in PALS, RVGLS, and increased LVEF
Dar et al.^18^	2018/USA	Prospective	66	N.A	TEE was performed before and after the LAAC.	LAA exclusion appears to improve the mechanical function of LA when assessed by STE.
Dippenaar et al.^19^	2021/South africa	Prospective	32	N.A	32 patients underwent LAAC. LA reservoir, conduit and contractile strain and strain rate were assessed with two‐dimensional speckle tracking echocardiography	No statistically significant improvement in LA mechanical function was seen after LAAO with the Amplatz or Amulet device, althougha trend towards improved strain was observed for reservoir and conduitstrain, with a worsening in contractile strain.

Abbreviations: LA, left atrial; LAA, left atrial appendage; LAAC, left atrial appendage closure; LV, left ventricle; PALS, peak atrial longitudinal strain.

**Table 2:** Baseline characteristics

STUDY	Age	Male	HTN	DM	CAD	CHF	Afib	CHA2DS2‐VASc score	HAS‐BLED score
Madeira et al.^12^	71 ± 9	63%	N.A	N.A	N.A	N.A	Permanent (75%)	5 [4–5]	3 [2–3]
							Persistent (6%)		
							Paroxysmal (19%)		
Yang et al.^13^	61.8 ± 7.9	66%	69%	14%	23%	N.A	Persistent AF: 46%	3 (2, 4)	3 (2, 3)
							Long‐standing persistent AF: 54%		
Ijuin et al.^14^	75 ± 68	67%	97%	40%	40%	85%	Permanent: 65%	4.4 ± 1.4	4.1 ± 0.9
							Paroxysmal: 34%		
Coisne et al.^15^	67.1 ± 12.2	51.5%	82%	33%	N.A	N.A	N.A	4.5 ± 1.37	3.4 ± 1
Murtaza et al.^16^	76 ± 6.9	60%	96%	32%	76%	36%	Persistent: 56%	5.0 ± 1.7	4.0 ± 1.5
							Paroxysmal: 44%		
Sharma et al.^17^	73.2 ± 9.0	70%	90%	48%	68%	65.7%	N.A	4.5 ± 1.3	N.A
Dar et al.^18^	70 ± 9.23	66%	82%	30%	48%	24%	Paroxysmal: 44%	3.7 ± 1.7	3.3 ± 1.4
							Persistent: 32%		
							Longstanding: 24%		
Dippenaar et al.^19^	N.A	N.A	N.A	N.A	N.A	N.A	N.A	N.A	N.A

Abbreviations: Afib, atrial fibrillation; CAD, coronary artery disease; CHF, congestive heart failure; DM, diabetes mellitus; HTN, hypertension.

### 3.2 Outcomes

**Figure 2:**
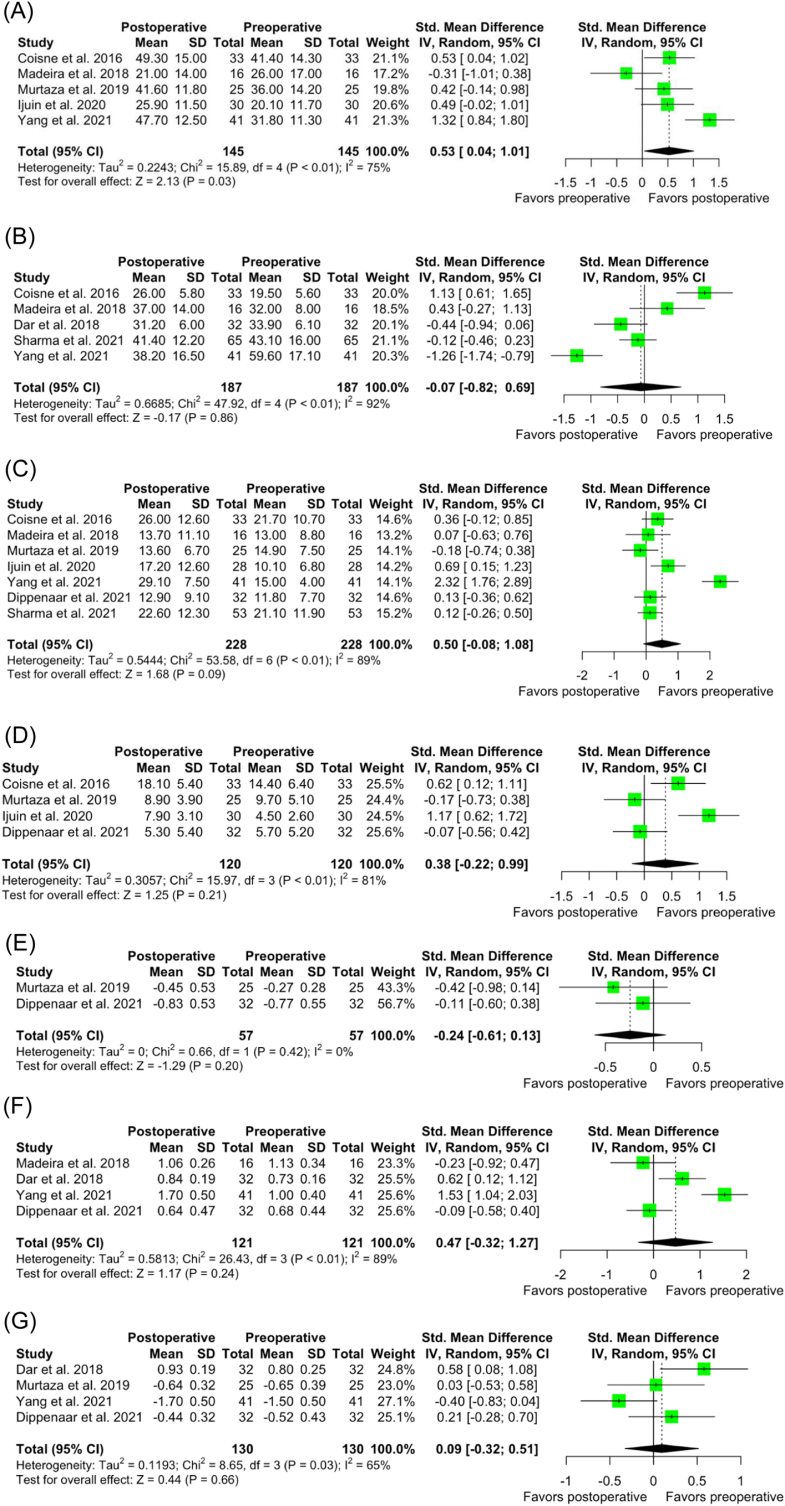
(A) Forest plot of TAEF. (B) Forest plot of LA volume. (C) Forest plot of PALS. (D) Forest plot of PACS. (E) Forest plot of the strain during atrial contraction. (F) Forest plot of the strain during ventricular systole. (G) Forest plot of the strain during ventricular diastole. CI, confidence interval; LA, left atrial; PACS, peak atrial contraction strain; TAEF, total atrial emptying fraction

There was significant increase in LA emptying fraction following LAAC compared with preoperative function (SMD: 0.53; 95% CI: 0.04–1.01; *p* = .03; *I* ^2^ = 75%) (Figure 2A). There was no significant difference following LAAC in terms of LA volume (SMD: −0.07; 95% CI: −0.82–0.69; *p* = .86; *I* ^2^ = 92%) PALS (SMD: 0.50; 95% CI: −0.08–1.08; *p* = .09; *I* ^2^ = 89%), PACS (SMD: 0.38; 95% CI: −0.22–0.99; *p* = .21; *I* ^2^ = 81%), strain during atrial contraction (SMD: −0.24; 95% CI: −0.61–0.13; *p* = .20; *I* ^2^ = 0%), strain during ventricular systole (SMD: 0.47; 95% CI: −0.32–1.27; *p* = .24; *I* ^2^ = 89%), strain during ventricular diastole (SMD: 0.09; 95% CI: −0.32–0.51; *p* = .66; *I* ^2^ = 65%) (Figure 2B–G).

### 3.3 Heterogeneity evaluation and sensitivity analyses

Heterogeneity was noted to be high in most of our studied outcomes. Baujat plots were utilized to evaluate this heterogeneity. Leave‐one‐out analyses were performed to detect any influential effects of the included studies, especially those with the highest contribution to heterogeneity.

− Total atrial emptying fraction (TAEF): Yang et al.^13^ was the study of most contribution to heterogeneity. Omitting Yang et al.^13^ dropped *I* ^2^ from 75% to 31% without significant changes to the results. Omitting Murtaza et al.,^16^ Ijuin et al.,^14^ or Coisne et al.^15^ makes the results nonsignificant (Figure S3A).
− LA volume: Coisne et al.^15^ was the study with most contribution to heterogeneity. However, with omitting it *I* ^2^ dropped only from 92% to 85%. Leave‐one‐out analyses did not show any significant changes in the results (Figure S3B).
− PALS: Yang et al.^13^ was also the study with the highest contribution to heterogeneity. When we excluded it, the *I* ^2^ dropped from 89% to 14%. Leave‐one‐out analyses of the studies evaluating this outcome did not show any significant changes of the results (Figure S3C).
− PACS: Yang et al.^13^ was also the study with the highest contribution to heterogeneity. When we excluded it, the *I* ^2^ dropped from 81% to 64%. Leave‐one‐out analyses of the studies evaluating this outcome did not show any significant changes in the results (Figure S3D).
− Strain during ventricular systole and diastole: Yang et al.^13^ was also the study with the highest contribution to heterogeneity. When we excluded it, the *I* ^2^ dropped from 89% to 63% for the ventricular systole and from 65% to 11% for the strain during ventricular diastole. Leave‐one‐out analyses of the studies evaluating these outcomes did not show any significant changes in the results (Figure S3F,G).

## 4 DISCUSSION

Our report investigates differences in LA function following LAAC in 8 studies including 260 patients. We report that following LAAC, significant improvements of LA reservoir function as assessed by LAEF occur, with no significant differences in LA reservoir function assessed by PALS or LA contractile function assessed by PACS.

LA function is a cyclic process of three major components; LA reservoir function, conduit function and contractile function. LA reservoir function, which entails the stretch of LA myocytes to accommodate pulmonary venous return during left ventricle (LV) systole, usually correlates with LA compliance. During ventricular diastole, the mitral valve opens with movement of blood from the LA to the LV, which reflects the conduit function of LA. LA contraction follows at the end of diastole, which represents the contractile function of LA.^20^, ^21^

Our study has demonstrated significant improvement in LA reservoir function assessed by LAEF after LAA closure. One possible etiology is increased venous return after LAAC since LAA plays a vital role in volume balance by regulating both atrial and BNPs. Majunke et al.^22^ compared levels of atrial natriuretic peptide (ANP) and BNP in 31 patients undergoing LAAC before and immediately after the procedure. They demonstrated a rapid decline of both ANP and BNP immediately after LAA exclusion and before discharge. Another plausible explanation by Höllmer et al. is the dwindle in maximal LA volume after LAA closure with subsequent elevation of atrial pressure and eventually an increase in LAEF. In other words, LAEF is inversely proportional to LA volume, which decreases after LAA exclusion.^23^ Tabata et al.^24^ demonstrated the impact of LAA surgical clamping on LA function on eight patients undergoing coronary artery bypass graft and surgical repair of severe mitral regurgitation. They denoted that LAA is more compliant than the LA proper, and its exclusion would ultimately shift the LA pressure curve upward and to the left. As a result, total LAEF would increase after LAA closure.

Total LAEF is identified as an important prognostic marker in various cardiac pathologies. For instance, Yang et al.^25^ conducted a retrospective study comparing LA function, including LAEF, between nonobstructive hypertrophic cardiomyopathy (NO‐HCM) and the control group with cardiac magnetic resonance. They concluded that LA functions, including total LAEF, are impaired in NO‐HCM even before LA starts to get enlarged. Moreover, LA deformation is heavily investigated in the heart failure population as LA represents a unique connection between LV and pulmonary circulation. An observational study conducted by Melenovsky et al.^26^ has revealed that total LAEF is a valid predictor of right ventricle function in patients with both heart failure reduced ejection fraction and heart failure with preserved ejection fraction (HFpEF).

Moreover, impaired LAEF in heart failure patients with concomitant atrial fibrillation is associated with worsening New York heart association classification. Intriguingly, impaired total LAEF in HFpEF patients was correlated with a higher mortality rate in those subsets of the population.^26^ In a randomized clinical trial by Alli et al.,^27^ LAA closure has shown to be superior to warfarin for quality of life at 12 months follow‐up. LAA was then sub‐grouped into warfarin naïve and non‐warfarin naïve, and surprisingly warfarin naïve patients showed better total physical score and physical functioning, which can be partially attributed to improved LA function as illustrated by our analysis.

Moreover, our study showed no difference in LA contractile function assessed by PACS before and after LAA exclusion. While Coisne et al.^15^ reported improved LA contractile function at 45 days follow‐up of LAA exclusion through Frank‐Starling mechanism. Our net analysis reported no difference in LA contractile function assessed by PACS, which can be tied to concomitant LA ischemia or fibrotic changes that impede LA contractility function.^28^ Exhausted frank starling mechanism in the setting of mitral regurgitation or chronic increase in preload is also a potential cause of lack of contractile function improvement after LAA closure which shifts LA pressure‐volume curve downward and to the left.^29^

Last, the Data included in our analysis exhibits significant heterogeneity, which might be due to the differences in the study population. Variable follow‐up durations have been identified as well. Different techniques for LAA closure have been used, including Watchman, Lariat, and ACP devices. More studies are needed to compare the efficacy of various LAAC devices on LA deformation.

## 5 CONCLUSION

LAA exclusion is associated with improvement of LA reservoir function assessed by TAEF. PALS and LA contractile function did not differ significantly after LAA exclusion. More research is warranted for better understanding of LA hemodynamics and its implications in clinical vignette.

## CONFLICTS OF INTEREST

The authors declare no conflicts of interest.

## Supporting information

Supporting information.

## Data Availability

The data that supports the findings of this study are available in the supplementary material of this article.
