# Supplementary material for: A systematic review and meta‐analysis of the impact of the left atrial appendage closure on left atrial function
Source: Clin Cardiol. 2022 Apr 2;45(6):614–21. doi: 10.1002/clc.23824 (PMC9175246; doi:10.1002/clc.23824)
Supplement: Supplementary file 1 — Supporting information. [file CLC-45-614-s001.pdf]

Supplementary Figure 3-A: Leave-one-out analysis and baujat plot of TAEF

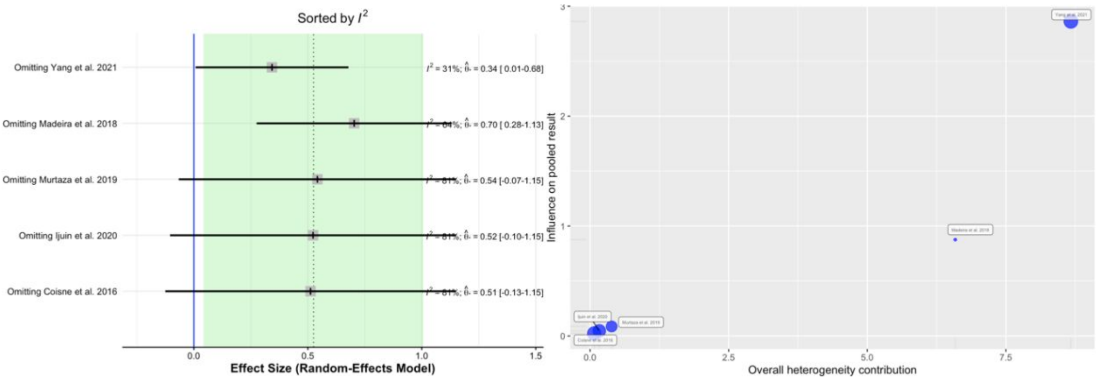


Supplementary Figure 3-B: Leave-one-out analysis and baujat plot of LA volume

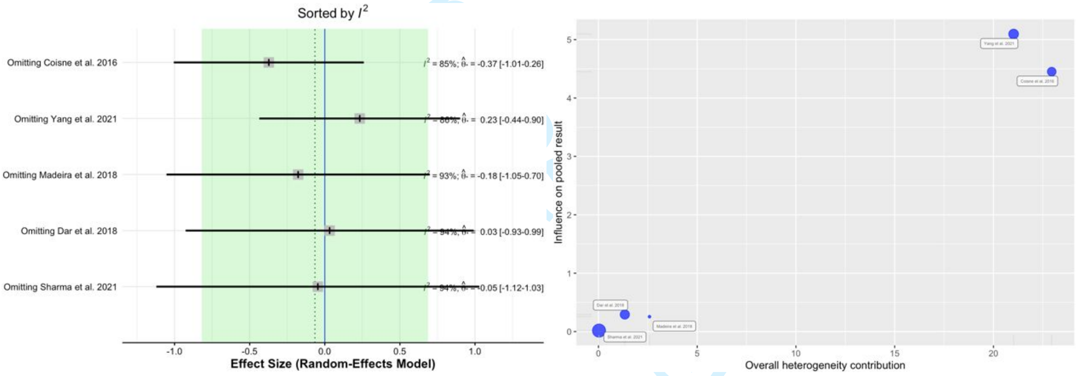


Supplementary Figure 3-C: Leave-one-out analysis and baujat plot of PALS

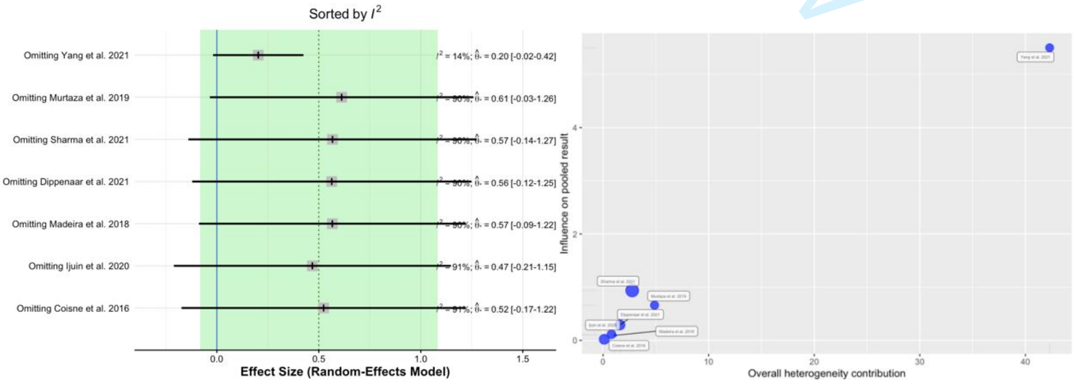


For Peer Review

Supplementary Figure 3-D: Leave-one-out analysis and baujat plot of PACS

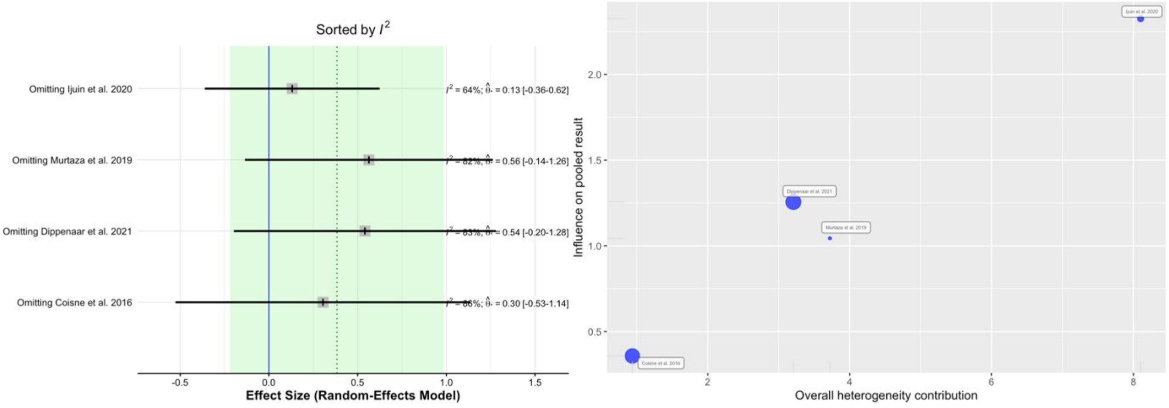


Supplementary Figure 3-E: Leave-one-out analysis and baujat plot of the strain during atrial contraction

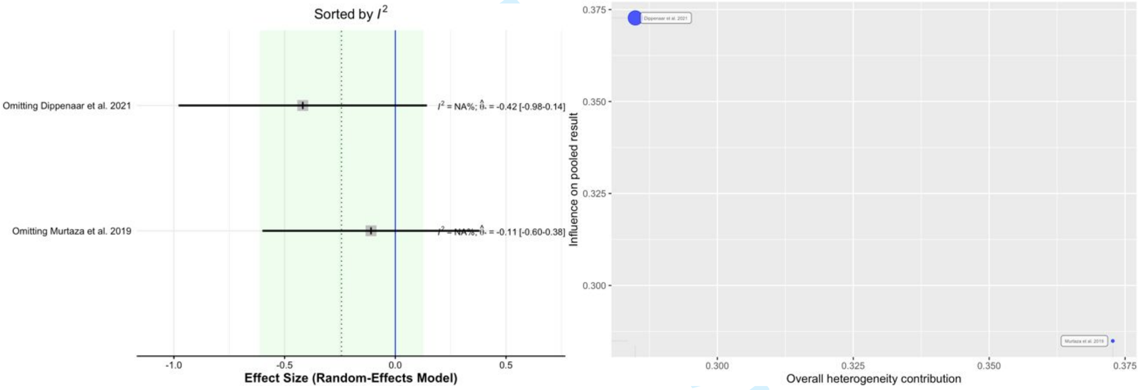


Supplementary Figure 3-F: Leave-one-out analysis and baujat plot of the strain during ventricular systole

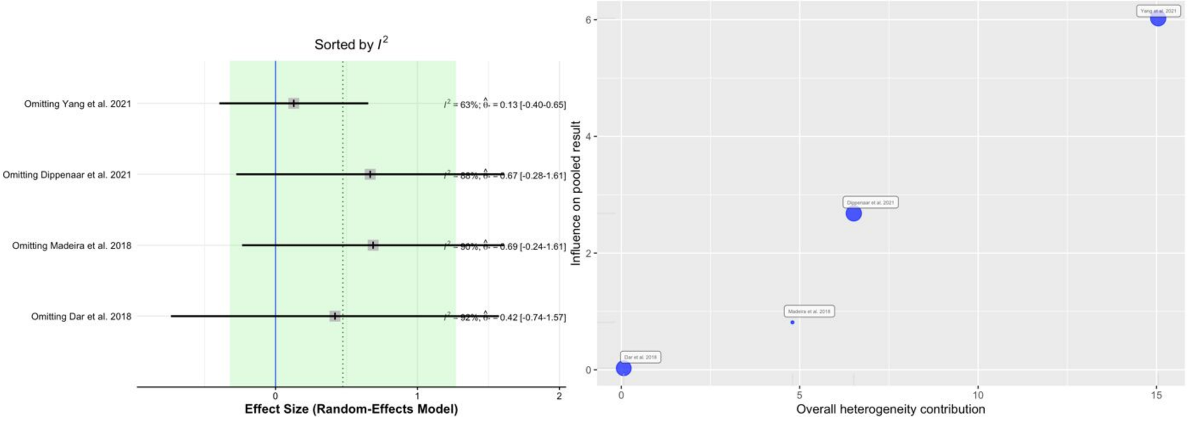

Supplementary Figure 3-G: Leave-one-out analysis and baujat plot of the strain during ventricular diastole

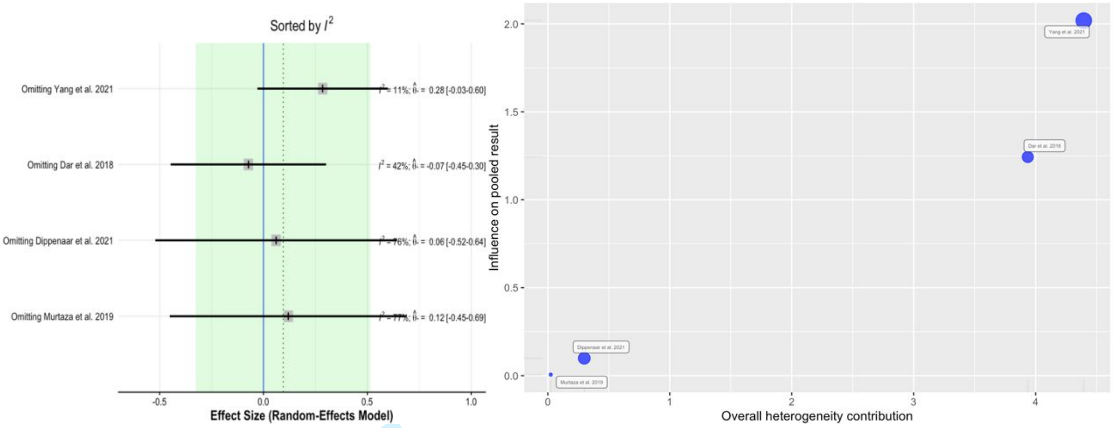


For Peer Review

Supplementary table 1:

Newcastle-Ottawa scale for Quality assessment of the included studies

| | Selection | | | | Comparability | | Outcome | | | |
|---|---|---|---|---|---|---|---|---|---|---|
| Study | Representativeness of exposed cohort | Selection of non-exposed cohort | Ascertainment of exposure | Demonstration that outcome of interest was not present at start of study | Adjust for the most important risk factors | Adjust for other risk factors | Assessment of outcome | Follow-up length | Loss to follow-up rate | **Total quality score** |
| Marta Madeira et al/2018 | 0 | 0 | 1 | 1 | 0 | 0 | 1 | 1 | 1 | **5** |
| Yang et al /2021 | 0 | 0 | 1 | 1 | 1 | 1 | 1 | 1 | 1 | **7** |
| Shun Ijuin et al /2020 | 0 | 0 | 1 | 1 | 0 | 0 | 1 | 1 | 1 | **5** |
| Augustin Coisne et al /2016 | 0 | 0 | 1 | 1 | 0 | 0 | 1 | 1 | 1 | **5** |
| Ghulam Murtaza et al/2019 | 0 | 0 | 1 | 0 | 0 | 0 | 1 | 0 | 1 | **3** |
| Esseim Sharma et al/2021 | 0 | 0 | 1 | 1 | 0 | 0 | 1 | 0 | 1 | **4** |
| Dar/2018 | 0 | 0 | 1 | 1 | 0 | 0 | 1 | 1 | 1 | **5** |
| Dippenaar/2021 | NA | NA | NA | NA | NA | NA | NA | NA | NA | **NA** |

# Tables

Table 1: summary of the included studies

| Study | Year/Country | Study design | Sample size | Follow up | Summary of methodology | Outcome |
|---|---|---|---|---|---|---|
| Marta Madeira et al | 2018/Portugal | Retrospective | 16 | N.A | TEE performed before and after LAAC. LA volumes were calculated using the biplane method, and LA mechanics were assessed using STE.. The analysis focused on the LA reservoir phase strain and strain rate. | percutaneous LAA closure does not affect LA reservoir function |
| Yang et al | 2021/China | Retrospective | 65 | 12 months | Intervention group: Combined AF ablation and LAAC.<br><br>Control group: AF ablation.<br><br>echocardiography and speckle tracking echocardiography were performed to assess LA reservoir, conduit, and contractile function | Both the combined therapy group and the simple ablation group demonstrated significant improvement in LA function. But most of the effects appeared to result from ablation, not LAAC |
| Shun Ijuin et al | 2020/Germany | Retrospective | 95 | 180 days | 95 patients who underwent percutaneous LAAC. LA strain was evaluated at three different time intervals by TEE (baseline, 45 days, and 180 days after the procedure | The study showed improvement in TEE-derived LA strain following LAAC within 45 days of implantation. |
| Augustin Coisne et al | 2016/France | Prospective | 33 | 45 days | 33 patients evaluated by TEE at time of discharge and 45 days. | LAA closure was associated with an improvement in LA mechanical function |
| Ghulam Murtaza et al | 2019/USA | Prospective | 25 | N.A | 25 patients underwent LAAC. LA function parameters | LAAC leads to improvement in all volumetric indices of the |

For Peer Review

| | | | | | | |
|---|---|---|---|---|---|---|
| | | | | | (volumetric, strain indices) were assessed by speckle tracking before and after | LA. However, there was discrepancy between volumetric and strain indices |
| Esseim Sharma et al | 2021/USA | Retrospective | 67 | N.A | TTE performed before and after LAAC. | LAAC resulted in elevated LV filing pressure, improvement in PALS, RVGLS, and increased LVEF |
| Dar et al | 2018/USA | Prospective | 66 | N.A | TEE was performed before and after the LAAC. | LAA exclusion appears to improve the mechanical function of LA when assessed by STE. |
| Dippenaar et al | 2021/South africa | Prospective | 32 | N.A | 32 patients underwent LAAC. LA reservoir, conduit and contractile strain and strain rate were assessed with two-dimensional speckle tracking echocardiography | No statistically significant improvement in LA mechanical function was seen after LAAO with the Amplatz or Amulet device, although a trend towards improved strain was observed for reservoir and conduit strain, with a worsening in contractile strain. |

For Peer Review

TABLE 2: baseline characteristics

| STUDY | Age | Male | HTN | DM | CAD | CHF | Afib | CHA2DS2-VASc score | HAS-BLED score |
|---|---|---|---|---|---|---|---|---|---|
| Marta Madeira et al | 71 ± 9 | 63% | N.A | N.A | N.A | N.A | Permanent (75%) Persistent (6%) Paroxysmal (19%) | 5 [4 - 5] | 3 [2 - 3] |

| | | | | | | | | | |
|---|---|---|---|---|---|---|---|---|---|
| Yang et al | 61.8 ± 7.9 | 66% | 69% | 14% | 23% | N.A | Persistent AF: 46%<br>Long-standing persistent AF: 54% | 3 (2, 4) | 3 (2, 3) |
| Shun Ijuin et al | 75±68 | 67% | 97% | 40% | 40% | 85% | Permanent: 65%<br>Paroxysmal: 34% | 4.4 ± 1.4 | 4.1± 0.9 |
| Augustin Coisne et al | 67.1 ± 12.2 | 51.5% | 82% | 33% | N.A | N.A | N.A | 4.5 ± 1.37 | 3.4 ± 1 |
| Ghulam Murtaza et al | 76 ± 6.9 | 60% | 96% | 32% | 76% | 36% | Persistent: 56%<br>Paroxysmal: 44% | 5.0 ± 1.7 | 4.0 ± 1.5 |
| Esseim Sharma et al | 73.2±9.0 | 70% | 90% | 48% | 68% | 65.7% | N.A | 4.5 ± 1.3 | N.A |
| Dar et al | 70 ± 9.23 | 66% | 82% | 30% | 48% | 24% | Paroxysmal: 44%<br>Persistent: 32%<br>Longstanding: 24% | 3.7 ± 1.7 | 3.3 ± 1.4 |
| Dippenaar et al | N.A | N.A | N.A | N.A | N.A | N.A | N.A | N.A | N.A |

For Peer Review

Supplementary table 1:

Newcastle-Ottawa scale for Quality assessment of the included studies

| | Selection | | | | Comparability | | Outcome | | | |
|---|---|---|---|---|---|---|---|---|---|---|
| Study | Representativeness of exposed cohort | Selection of non-exposed cohort | Ascertainment of exposure | Demonstration that outcome of interest was not present at start of study | Adjust for the most important risk factors | Adjust for other risk factors | Assessment of outcome | Follow-up length | Loss to follow-up rate | **Total quality score** |
| Marta Madeira et al/2018 | 0 | 0 | 1 | 1 | 0 | 0 | 1 | 1 | 1 | **5** |
| Yang et al /2021 | 0 | 0 | 1 | 1 | 1 | 1 | 1 | 1 | 1 | **7** |
| Shun Ijuin et al /2020 | 0 | 0 | 1 | 1 | 0 | 0 | 1 | 1 | 1 | **5** |
| Augustin Coisne et al /2016 | 0 | 0 | 1 | 1 | 0 | 0 | 1 | 1 | 1 | **5** |
| Ghulam Murtaza et al/2019 | 0 | 0 | 1 | 0 | 0 | 0 | 1 | 0 | 1 | **3** |
| Esseim Sharma et al/2021 | 0 | 0 | 1 | 1 | 0 | 0 | 1 | 0 | 1 | **4** |
| Dar/2018 | 0 | 0 | 1 | 1 | 0 | 0 | 1 | 1 | 1 | **5** |
| Dippenaar/2021 | NA | NA | NA | NA | NA | NA | NA | NA | NA | **NA** |

For Peer Review
